# Case Report: Deep and durable response to talazoparib in germline *BRCA2*-mutated rectal neuroendocrine carcinoma

**DOI:** 10.3389/fonc.2025.1705579

**Published:** 2025-12-19

**Authors:** Milan Khealani, Byoung Uk Park, Robben Schat, Emmanuel S. Antonarakis, Arjun Gupta

**Affiliations:** 1 Division of Hematology, Oncology & Transplantation, University of Minnesota, Minneapolis, MN, United States; 2 Department of Laboratory Medicine and Pathology, University of Minnesota, Minneapolis, MN, United States; 3 Department of Radiology, University of Minnesota, Minneapolis, MN, United States

**Keywords:** rectal neuroendocrine carcinoma, gBRCA2 mutation, talazoparib, PARP inhibitor, case report

## Abstract

High-grade neuroendocrine carcinoma (NEC) of the rectum is a rare and aggressive malignancy, with limited treatment options and a poor prognosis. We report the successful off-label use of the PARP inhibitor talazoparib in a patient with metastatic, germline *BRCA2*-mutated rectal NEC who had a contraindication to standard immunotherapy due to underlying autoimmune disease. A 55-year-old man with ongoing severe psoriatic arthritis presented with a two-week history of rectal pain, abdominal distention, and diarrhea. Cross-sectional imaging demonstrated a rectal mass with mesorectal lymphadenopathy and multiple liver metastases. Biopsy of the rectal lesion demonstrated a high-grade, poorly differentiated NEC with a Ki-67 proliferation index of 99%. Comprehensive tumor molecular profiling identified a pathogenic *BRCA2* mutation (c.5291C>G; p.Ser1764*, with loss of the wild-type allele), which was confirmed to be a germline alteration through germline testing. There were also biallelic inactivations of *APC*, *TP53*, and *RB1*. The patient received four cycles of induction chemotherapy with carboplatin and etoposide, achieving a partial radiographic response; however, treatment was complicated by cytopenias and significant fatigue. Immunotherapy was considered inappropriate as part of his initial systemic therapy regimen or as maintenance treatment due to the severe underlying autoimmune condition. Based on the germline *BRCA2*-mutated (g*BRCA*) status, we requested emergency approval for off-label use of talazoparib, a poly(ADP ribose) polymerase (PARP) inhibitor. At 12.5 months from initial diagnosis, including after 7.5 months on talazoparib, the patient continues to show ongoing radiographic response with excellent tolerability and no adverse effects. This case illustrates the potential role of PARP inhibitors in the management of *BRCA2*-mutated high-grade rectal NEC. Molecular profiling techniques may uncover actionable genetic targets in rare, aggressive cancers without standard treatment options or in patients with co-morbidities that preclude standard treatment regimens.

## Introduction

High-grade neuroendocrine carcinoma (HGNEC) of the rectum is a rare and aggressive malignancy, accounting for fewer than 1-2% of all gastrointestinal malignancies (1, 2). These poorly differentiated tumors, which can include both large cell and small cell subtypes, are characterized by high mitotic rates, frequent metastatic spread at diagnosis, and poor prognosis (1). Median survival for rectal small-cell NEC ranges from 7 to 11 months, with five-year survival rates of 8-15% (3).

Given the rarity of these neoplasms, there is a lack of prospective data to guide management, and best practices have historically been extrapolated from protocols for pulmonary HGNECs (4). However, recently published expert consensus recommendations from the North American Neuroendocrine Tumor Society (NANETS) now provide updated guidance for gastroenteropancreatic HGNECs. For metastatic disease, first-line management consists of platinum-based chemotherapy, typically in combination with etoposide or irinotecan (5, 6).

Immune checkpoint inhibitors (ICIs) have shifted the treatment paradigm in analogous tumors, most notably in extensive-stage small cell lung cancer (SCLC). The addition of a PD-L1 inhibitor, such as atezolizumab or durvalumab, to first-line platinum-etoposide chemotherapy demonstrated a significant overall survival benefit in SCLC, establishing chemo-immunotherapy as the new standard of care (7, 8). Although similar overall survival data do not yet exist for GI NECs, this SCLC paradigm has been widely adopted, and first-line chemo-immunotherapy is often considered for these patients in the absence of other compelling options. In the non-pancreatic neuroendocrine tumor cohort of the DART trial, ipilimumab plus nivolumab produced meaningful response rates, but outcomes remained modest overall (9). However, in patients with pre-existing autoimmune conditions, ICIs may be associated with a heightened risk of disease flares and immune-related adverse events (irAEs) (10–12).

The present case report describes a patient with metastatic high-grade rectal NEC and severe psoriatic arthritis, in whom immunotherapy was considered high risk and was deferred. Following initial treatment with carboplatin and etoposide, the patient underwent comprehensive molecular profiling, which revealed a pathogenic germline *BRCA2* mutation (c.5291C>G; p.Ser1764*) with loss of the wild-type allele, for which clinically available therapeutic targets exist, including PARP inhibitors.

While germline *BRCA2* mutations are well-established drivers of breast, ovarian, prostate, and pancreatic cancers (13), their role in neuroendocrine carcinoma remains less defined, although they have been reported in prostate, gallbladder, and ovarian NECs (14–16).

The efficacy of PARP inhibitors in germline BRCA-mutated cancers is well established in breast, ovarian, prostate, and other gastrointestinal malignancies, including pancreatic adenocarcinoma (17–23). Guided by these data and isolated reports of success in BRCA2-mutated small-cell carcinoma of the cervix and neuroendocrine prostate cancer (5, 14, 24), we pursued off-label treatment with the PARP inhibitor talazoparib.

To our knowledge, this approach has not previously been described in the context of rectal NEC. This case highlights the potential for personalized therapy with a strong biological rationale in rare and treatment-resistant cancers and adds to the emerging evidence supporting PARP inhibition in *BRCA2*-mutated HGNEC.

## Case description

A 55-year-old male with a past medical history of severe polyarticular psoriatic arthritis, obesity, type 2 diabetes mellitus, and hyperlipidemia presented to his primary care physician with a two-week history of rectal pain, abdominal distension, and a sensation of rectal tenesmus. He also reported alternating constipation and diarrhea and denied bleeding, melena, fever, or weight loss. He had no prior history of gastrointestinal malignancy, inflammatory bowel disease, or other colorectal conditions. His family history was notable for prostate cancer in a paternal grandfather and breast cancer in a paternal aunt. On physical examination, the digital rectal exam revealed a left anterior ulcerated, fixed mass just above the anorectal ring, fixed to the prostate but not to the anal sphincter complex. The sphincter tone was preserved.

Based on his presenting symptoms and history of psoriatic arthritis, the initial clinical suspicion was for a perianal abscess or fistula related to inflammatory bowel disease. Of note his psoriatic arthritis was controlled on long-standing adalimumab and methotrexate.

### Timeline

The timeline of relevant clinical events, interventions, and outcomes is summarized in **Table 1**.

**Table 1:** Timeline of key clinical events.

DATE	EVENT
Month 0	Diagnosis: Patient presents with rectal symptoms. Biopsy confirms high-grade rectal neuroendocrine carcinoma (Ki-67 99%).
Month 1	Staging & Genomics: Imaging confirms metastatic disease (T4N2M1) with extensive liver involvement. Germline testing reveals a pathogenic *gBRCA2*mutation.
Months 1-3	First-Line Chemotherapy: Patient completes four cycles of carboplatin and etoposide, achieving a partial response.
Month 4	Treatment Pivot: Further chemotherapy is halted due to fatigue and cytopenias. A decision is made to switch to targeted therapy based on the *gBRCA2* mutation.
Month 5	Targeted Therapy Initiated: Maintenance therapy with the PARP inhibitor talazoparib (0.5 mg daily) is started.
Months 7-11	Response Monitoring: Serial imaging confirms a progressive and deep response to talazoparib.
Month 12.5 (7.5 months into therapy)	Confirmation of Durable Response: MRI at ~9 months of therapy confirms a near-complete and durable response.
Present	Ongoing Control: Patient continues on maintenance talazoparib with excellent tolerability and sustained disease control.

### Diagnostic assessment

**Figure 1:**
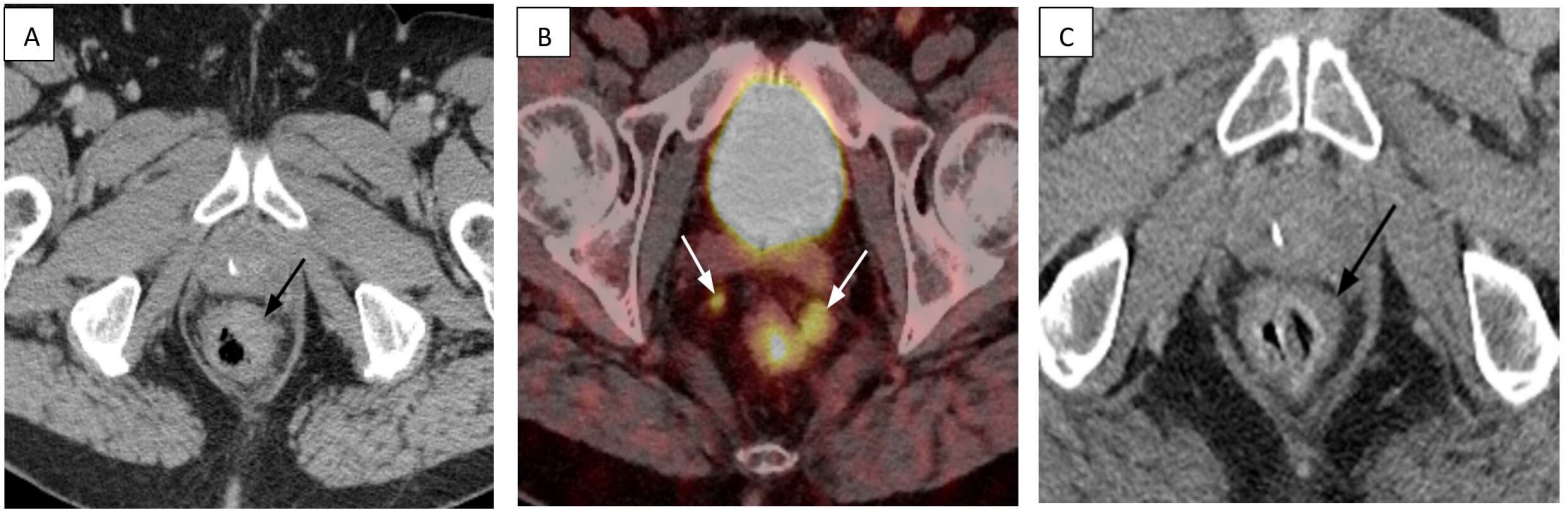
CT and PET/CT imaging of the primary rectal tumor and regional lymph nodes before and after treatment. **(A)** Baseline axial contrast-enhanced CT scan (July 2024) demonstrates a large, semi-annular soft tissue mass in the low anterior left rectum (black arrow). **(B)** Staging Gallium-68 dotatate PET/CT (July 2024) reveals dotatate-avid mesorectal lymphadenopathy superior to the primary mass (white arrows). **(C)** Follow-up axial contrast-enhanced CT scan (March 2025), after systemic therapy, shows a significant treatment response with only minimal residual wall thickening at the tumor site (black arrow) and complete resolution of the lymphadenopathy.

**Figure 2:**
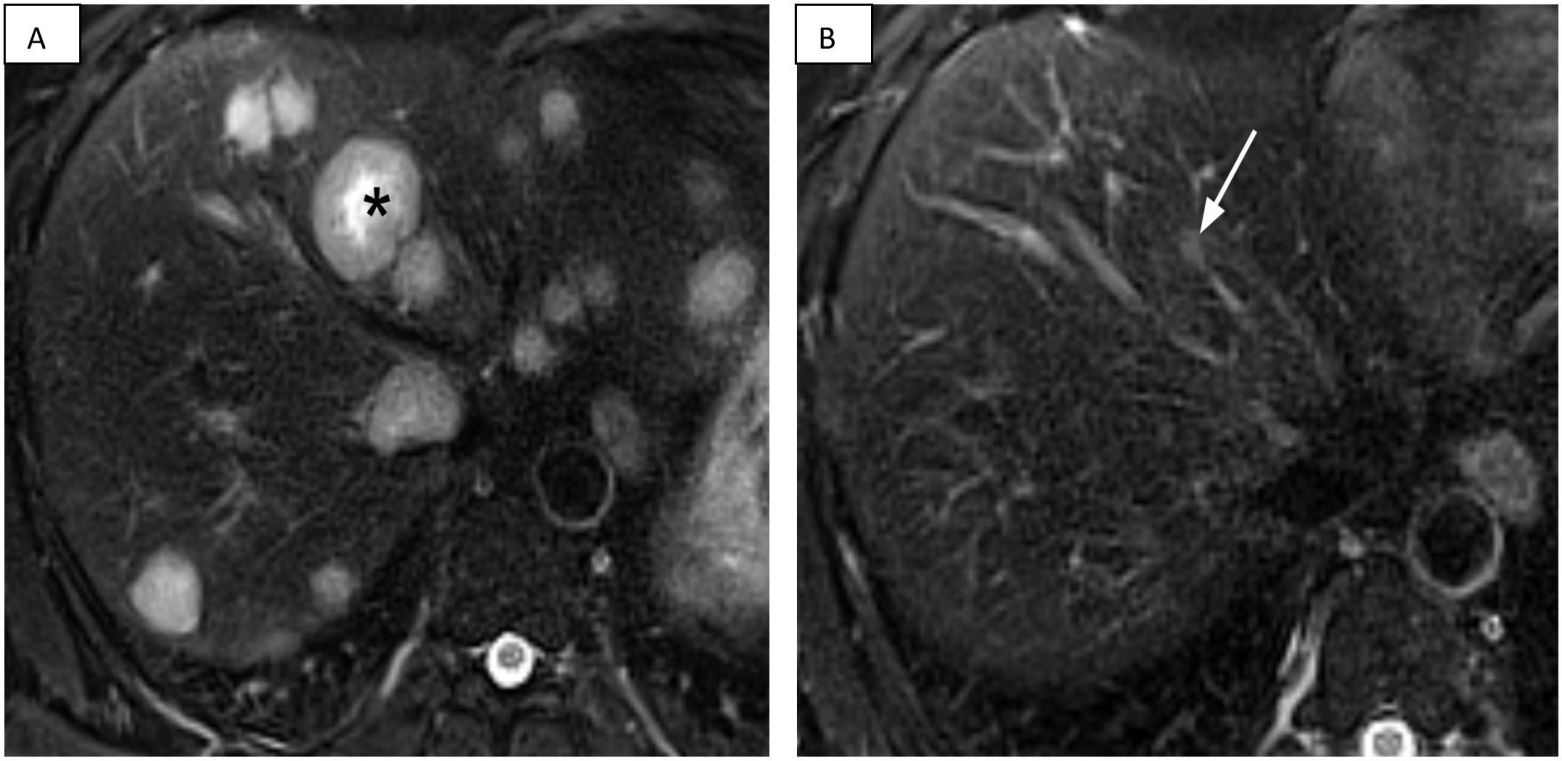
MRI of the abdomen. **(A)** Baseline MRI of the abdomen. Axial T2-weighted fat suppressed MRI of the abdomen at baseline (August 2024) reveals innumerable, widespread T2-hyperintense hepatic metastases involving all segments of the liver (black asterisk denotes largest lesion). **(B)** Follow-up MRI of the abdomen. A follow-up axial T2-weighted fat suppressed MRI of the abdomen (August 2025), after approximately 7.5 months of talazoparib, demonstrates near resolution of the hepatic metastases, with only minimal residual signal abnormality within some of the initially largest lesions (white arrow).

Initial evaluation included contrast-enhanced computed tomography (CT) of the abdomen and pelvis, which revealed asymmetric thickening of the distal left anterolateral rectal wall and associated mesorectal lymphadenopathy as well as numerous hepatic lesions (**Figures 1A**, **2A**). There was no evidence of perirectal or perianal abscess or fistula, prompting further evaluation for a suspected rectal neoplasm.

**Figure 3:**
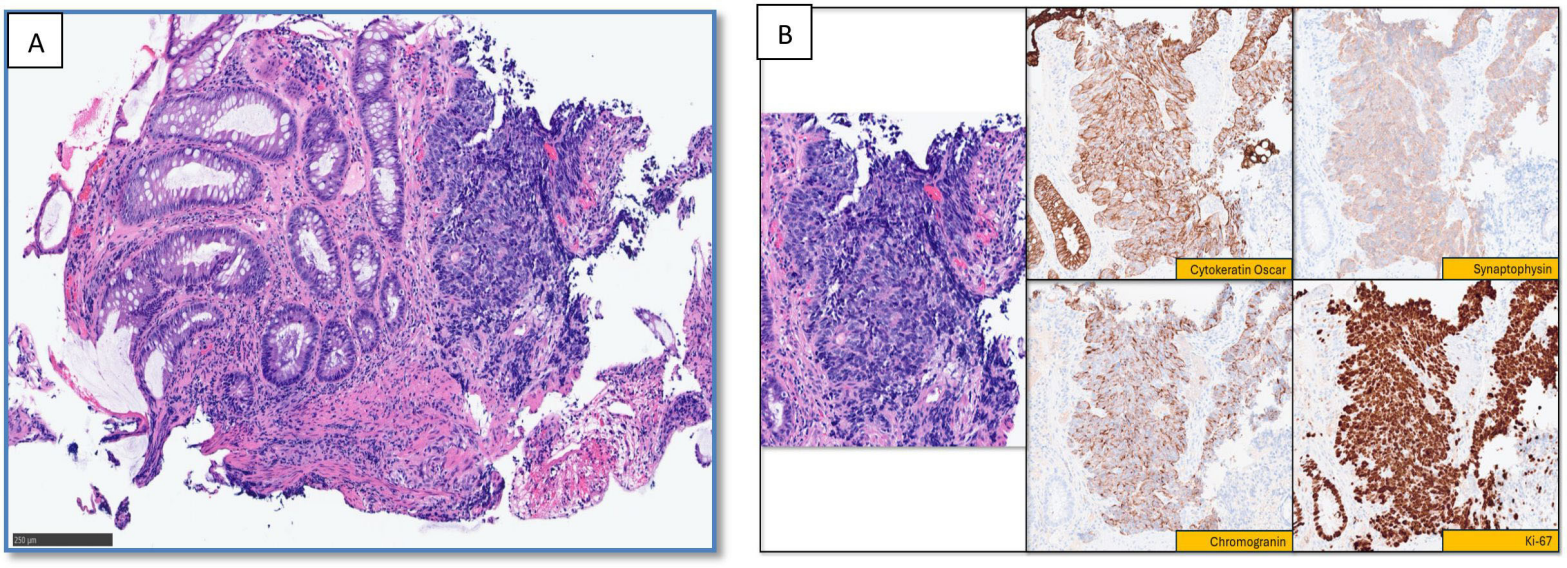
Histopathology and immunohistochemical staining of the rectal neuroendocrine carcinoma. **(A)** Microscopic image of the biopsied colonic mucosa (left) showing a proliferation of atypical cells arranged in a solid architectural pattern (right). The cells exhibit high nuclear-to-cytoplasmic (N: C) ratios, scant cytoplasm, hyperchromatic nuclei, and prominent nuclear molding. **(B)** Higher-power magnification of the area of interest, demonstrating sheets of small ovoid cells with scant cytoplasm. Limited immunohistochemical analysis shows tumor cell positivity for CK Oscar, synaptophysin, and chromogranin, supporting epithelial neuroendocrine neoplasm. The Ki-67 proliferation index is markedly elevated, approaching 100%. The histomorphology and immunohistochemical profile are consistent with a diagnosis of high-grade, poorly differentiated neuroendocrine carcinoma. .

Flexible sigmoidoscopy identified a 2.5 cm ulcerated mass at the left anterior aspect of the distal rectum, just above the dentate line. The biopsy of the lesion confirmed an invasive, high-grade, poorly differentiated NEC with a very high Ki-67 proliferation index of 99% (**Figures 3A, B**).

Further staging included a Gallium-68 DOTATATE PET/CT, which demonstrated modest radiotracer uptake in the rectal lesion (SUVmax 8.2) and several mesorectal lymph nodes (SUVmax 6.6), corresponding to a Krenning score of 2 (**Figure 1B**). Rectal MRI revealed a T4N2 semi-annular, ulcerating mass with invasion of the mesorectal fascia, the left prostatic neurovascular bundle, and the left seminal vesicle. An MRI of the liver revealed innumerable hepatic metastases. Laboratory studies revealed normal carcinoembryonic antigen (CEA) at 1.1 ng/mL (reference range ≤5.0 ng/mL) and chromogranin A at 110 ng/mL (reference range 0–187 ng/mL).

Given the aggressive nature of his disease and a family history of prostate and breast cancer, comprehensive molecular profiling and germline testing were pursued. Tumor profiling identified pathogenic somatic mutations in *BRCA2* (exon 11, p.S1764, with loss of the wild-type allele) and *TP53* (exon 5, p.V157F, with loss of the wild-type allele). Notably, the genome-wide loss-of-heterozygosity (gLOH) score was markedly elevated at 21%, suggesting homologous recombination deficiency (HRD). The tumor also showed bi-allelic inactivation of *APC* (p.L1511fs*; p.C599*), supporting a colorectal primary, and a homozygous deletion of the *RB1* gene. Additional findings included intact microsatellite stability, low tumor mutational burden (6 mutations/megabase), and HER2 negativity by immunohistochemistry (0+), as well as PD-L1 negativity (combined positive score: 0). No alterations were detected in *BRAF, KRAS, ALK, NTRK, RET, CDKN2A*, or *CDKN2B*. Germline testing confirmed the *BRCA2* variant (c.5291C>G; p.Ser1764*) as a pathogenic germline alteration consistent with hereditary breast and ovarian cancer syndrome, with no pathogenic *BRCA1* mutation detected.

Overall, the findings were consistent with high-grade, poorly differentiated rectal NEC with hepatic metastases (T4N2M1). Given the presence of metastatic disease, a very high Ki-67 proliferation index, and rapid clinical progression, these features indicated biological aggressive behavior.

## Treatment and outcomes

A multidisciplinary tumor board recommended first-line platinum–etoposide chemotherapy, consistent with small-cell lung cancer–based protocols. The patient received four cycles of carboplatin and etoposide. After cycle 2, he was incidentally found to have a subsegmental pulmonary embolism and started on apixaban. Following four cycles of platinum-based chemotherapy, CT imaging demonstrated a partial radiographic response. The dominant hepatic lesions decreased in size by approximately 90%, and mesorectal lymphadenopathy showed interval reduction with near resolution. No new metastatic lesions were identified. By the end of four cycles of combination chemotherapy, the patient had developed significant chemotherapy-related cytopenias and marked fatigue. In light of these cumulative toxicities, the decision was made to hold further chemotherapy.

Due to the patient’s severe psoriatic arthritis and the limited efficacy of ICIs in HGNEC, immunotherapy was not selected for maintenance. Given the germline BRCA2 mutation, suggestive family history, and limited benefit of prolonged cytotoxic chemotherapy, we obtained compassionate use approval for off-label talazoparib, a PARP inhibitor approved for germline BRCA-mutated breast and prostate cancers.

Given the patient’s significant chemotherapy-associated cytopenias, talazoparib was initiated at a reduced dose of 0.5 mg daily (standard starting dose 1 mg daily), in line with approved dose modification schemes and to mitigate the risk of recurrent marrow toxicity. Given his excellent tolerance and radiographic response at this dose, dose escalation was not required, and therapy was continued at 0.5 mg daily.

The patient’s baseline CT and MRI scans prior to chemotherapy demonstrated an infiltrative primary rectal mass with mesorectal lymphadenopathy (**Figure 1A**) and extensive hepatic metastatic disease burden with at least 50 lesions (**Figure 2A**).

Serial imaging documented a progressive and deep response. A CT scan at one month after initiation of chemotherapy showed reduction in all hepatic lesions by about 80%, decreased visibility of the rectal mass by CT, and approximate 50% reduction in mesorectal lymphadenopathy. A subsequent CT scan at three months, immediately before initiation of talazoparib, demonstrated further lesion regression with resolution of many of the previously seen hepatic metastases, greater than 90% reduction of the still visible hepatic lesion, nearly resolved mesorectal lymphadenopathy, and minimal residual rectal wall thickening at the site of the primary lesion. By the seven-month CT scan, the primary tumor was no longer visible by CT (**Figure 1C**) with resolution of mesorectal lymphadenopathy. A nine-month CT scan confirmed a progressive reduction in hepatic lesions compared to baseline with resolution of all but approximately 5–10 lesions and greater than 95% reduction from baseline of the still visible lesions. The most recent MRI–performed approximately 11 months after initiation of chemotherapy and 7.5 months after starting talazoparib–demonstrated a durable near-complete response with stable minimal residual visible disease (**Figure 2B**). No new or enlarging lesions were identified throughout the treatment course.

Throughout maintenance therapy with talazoparib, the patient maintained an ECOG performance status of 0, continued working full-time, and remained socially active. He has had no recurrent cytopenias. Given the near-complete radiographic response and excellent tolerability, we did not escalate talazoparib to 1 mg daily.

## Discussion

To our knowledge, this is the first report of a profound and durable response to a PARP inhibitor in a patient with germline *BRCA2*-mutated rectal NEC. While the use of PARP inhibitors has been described in isolated cases of cervical and prostate NECs, this is the first such report in a neuroendocrine tumor of gastrointestinal origin. This outcome underscores the critical importance of comprehensive genomic profiling in rare, high-grade malignancies, as it can uncover highly effective, non-standard therapeutic avenues, particularly when comorbidities limit conventional options. Our case demonstrates that when biallelic inactivation of *BRCA2* is present, PARP inhibition can be a transformative therapy, even in a historically treatment-resistant histology.

PARP inhibitors are oral targeted agents that exploit synthetic lethality in cancers with defective DNA damage repair. In tumors with biallelic *BRCA1/2* inactivation, homologous recombination (HR) is impaired; pharmacologic PARP inhibition disables PARP-mediated single-strand break/base-excision repair and promotes PARP trapping on DNA, leading to replication-fork collapse, accumulation of lethal DNA damage, and selective tumor-cell death in HRD tumors (25). This mechanism underpins their landmark approvals in *BRCA*-mutated breast, ovarian, pancreatic, and prostate cancers (17, 19, 21, 22, 26, 27). Given class-associated hematologic toxicities (cytopenias) and fatigue, close monitoring is warranted; in our patient, reduced-dose talazoparib was well tolerated with stable counts.

HGNEC of the rectum is rare, accounting for fewer than 1–2% of all gastrointestinal neuroendocrine neoplasms (1, 2). With HGNEC, there is a marked tendency toward early metastatic spread, most commonly involving the liver (28). Due to the low incidence of rectal NEC, there are no disease-specific treatment guidelines, and clinical management is typically adapted from small-cell lung carcinoma protocols, where standard first-line regimens often include platinum-based chemotherapy in combination with immunotherapy followed by immunotherapy maintenance.

The role of ICIs in high-grade extrapulmonary NECs has been an area of increasing interest, though clinical benefit has been modest. For example, the combination of ipilimumab and nivolumab demonstrated a response rate exceeding 40% in the DART trial’s non-pancreatic NEC cohort, with a median overall survival of 11 months (9). However, this level of activity has not been sufficient to establish ICIs as a standard or highly effective treatment, with most studies suggesting limited efficacy (29, 30). In our patient, severe psoriatic arthritis and the absence of predictive biomarkers (low TMB, PD-L1 negative) further weighed against immunotherapy.

In patients with pre-existing autoimmune disease, ICIs can increase the risk of autoimmune flares and immune-related adverse events, sometimes requiring immunosuppression or treatment interruption (30, 31).

Psoriatic arthritis involves both skin and joints, and flares can manifest in either domain, necessitating that ICIs should only be used in this context where there is adequate monitoring and management (32). Consensus guidelines support ICI use in non–life-threatening autoimmune disease with careful monitoring, whereas life-threatening disease warrants a case-by-case risk–benefit discussion and consideration of alternatives (33). Accordingly, we prioritized chemotherapy and pursued molecular profiling to identify non-immunotherapy options, which revealed a germline BRCA2 mutation.

Of additional interest, this patient’s psoriatic arthritis entered sustained clinical remission after discontinuation of biologic and disease-modifying therapy at the time of cancer diagnosis and has remained quiescent throughout his oncologic treatment. At present, it is not possible to determine whether this trajectory reflects an indirect effect of cancer-directed therapy, changes in the immune milieu associated with advanced malignancy and its treatment, or a coincidental fluctuation in autoimmune disease activity. There are insufficient data to support a causal role for PARP inhibition in modifying psoriatic arthritis, and any such relationship in this case remains speculative.

From a broader perspective, this case also illustrates how concomitant autoimmune disease can significantly constrain the use of immunotherapy and influence treatment sequencing in high-grade NEC. For patients with clinically significant autoimmune conditions, early comprehensive genomic profiling may help identify non-immunotherapy systemic options, such as PARP inhibition in the presence of BRCA2 or other HRD-associated alterations. While a single case cannot establish a treatment standard, our experience supports considering targeted approaches within a multidisciplinary framework when conventional chemo-immunotherapy strategies carry a high risk of autoimmune toxicity.

HGNECs are characterized by frequent TP53 and RB1 alterations (34). BRCA2 involvement in digestive HGNECs (e.g. colorectal or pancreatic NECs) appears rare: in a multicenter genomic analysis of 229 gastrointestinal high-grade neuroendocrine neoplasms, BRCA1/2 mutations were identified in only 5.3% of NECs (10/188 cases), and none of the variants tested were germline (35). In our patient, a pathogenic germline nonsense BRCA2 variant (c.5291C>G; p.Ser1764) with loss of the wild-type allele established biallelic inactivation and HRD, providing a strong rationale for PARP inhibition. This strategy is consistent with the Phase 2 TALAPRO-1 trial, in which talazoparib monotherapy produced durable anti-tumor activity in metastatic prostate cancer with BRCA alterations (36).

Although BRCA2-mutated NECs are uncommon, several case reports describe sustained responses to PARP inhibition across pancreatic, cervical, and prostatic BRCA2-mutated NECs, with disease control ranging from many months to several years (5, 14, 23, 27).

Although evidence is limited to individual cases and small series, these reports suggest that *BRCA2*-mutated high-grade NECs may exhibit meaningful sensitivity to PARP inhibition. Our therapeutic rationale was further supported by robust evidence in other *BRCA*-mutated malignancies. In addition, a recent phase II basket study of talazoparib in advanced solid tumors with diverse DNA damage repair alterations, including germline and somatic BRCA1/2 and other homologous recombination genes, demonstrated clinical benefit in a subset of patients, further supporting the concept of targeting HRD beyond traditional tumor types (37). In breast cancer, the phase III OlympiAD and EMBRACA trials demonstrated significantly improved progression-free survival with olaparib and talazoparib, respectively, compared to standard chemotherapy in patients with HER2-negative, germline *BRCA1/2*-mutated metastatic disease (19–21). The radiographic response pattern observed in this case—early improvement followed by continued deepening over time—is broadly consistent with reported talazoparib activity in germline BRCA-mutated breast cancer, where objective responses typically emerge within the first few months of therapy and can remain durable over many months (21). In prostate cancer, the PROfound and TALAPRO-2 trials demonstrated that PARP inhibitors significantly improve outcomes in patients with metastatic castration-resistant disease and homologous recombination repair alterations, with the most pronounced benefit observed in those with *BRCA1/2* mutations (17, 38).

Although our patient remains in radiographic remission on talazoparib, acquired resistance to PARP inhibitors is well described and may ultimately limit the duration of benefit. Reported mechanisms include restoration of homologous recombination through BRCA1/2 reversion or other HR pathway alterations, protection of stalled replication forks, alterations that reduce PARP1 trapping on DNA, and increased drug efflux via ATP-binding cassette transporters. These resistance pathways, summarized in a recent review of PARP inhibitor biology (39), are important to consider when counselling patients and planning subsequent treatment if and when progression occurs.

In view of the above evidence and the nature of the patient’s germline *BRCA2* mutation, we sought and obtained emergency FDA approval for talazoparib, an oral PARP inhibitor approved for germline *BRCA2*-mutated breast and prostate cancers. This resulted in a profound clinical benefit with preservation of quality of life and patient well-being.

## Patient perspective

From the patient’s perspective, this therapeutic journey has been life-changing. He began systemic therapy with considerable apprehension and found that, while first-line chemotherapy relieved his rectal symptoms, it left him too fatigued and cytopenic to continue his physically demanding construction work. The possibility of needing long-term intravenous chemotherapy was deeply concerning.

The transition to oral talazoparib marked a turning point. He described the daily pill as “like taking an aspirin,” with no noticeable side effects. Within about two months, he returned to full-time heavy labor, working more than 40 hours per week and reporting that it felt “great to be 100% normal.” He felt that this regimen restored his health, independence, and family life.

He has since become a strong advocate for molecular testing and personalized treatment strategies. He also notes that his severe psoriatic arthritis has remained in remission despite discontinuation of biologic and disease-modifying agents at the start of cancer therapy, and he has not required resumption of arthritis medications.

## Conclusion

To our knowledge, this is the first reported case of a patient with g*BRCA2*-mutated rectal NEC successfully treated with a PARP inhibitor as a result of strong underlying biological rationale. This case highlights the critical importance of germline and somatic testing for DNA damage repair gene alterations in patients with high-grade neuroendocrine neoplasms, as identifying a targetable mutation can expand therapeutic avenues and lead to sustained disease control. This outcome strongly supports broader molecular testing in this population and suggests that future studies, perhaps through basket trials targeting specific mutations, are warranted to formally evaluate the efficacy of PARP inhibition in this rare but molecularly-defined subset of patients.

## Data Availability

The data analyzed in this study is subject to the following licenses/restrictions: The data analyzed in this article are not publicly available due to patient confidentiality and institutional privacy policies. The authors can provide de identified data upon reasonable request and with appropriate ethical approvals. Requests to access these datasets should be directed to Arjun Gupta, arjgupta@umn.edu.
